# SnO_2_NPs as a Nontoxic Antiviral Agent for Designing Protective Masks against Human Coronavirus Infection

**DOI:** 10.1021/acsabm.5c00173

**Published:** 2025-04-09

**Authors:** Anna Baranowska-Korczyc, Dorota Kowalczyk, Marcin Chodkowski, Kamil Sobczak, Małgorzata Krzyżowska, Małgorzata Cieślak

**Affiliations:** † Department of Chemical Textiles Technologies, Łukasiewicz Research Network—Lodz Institute of Technology, M. Skłodowskiej-Curie Street 9/27, 90-570 Lodz, Poland; ‡ Military Institute of Hygiene and Epidemiology, Kozielska 4, 01-163 Warsaw, Poland; § University of Warsaw, Biological and Chemical Research Centre, University of Warsaw, Żwirki i Wigury 101, 02-089 Warsaw, Poland

**Keywords:** silk, silk fabric, SnO_2_, protective masks, antiviral properties, COVID-19

## Abstract

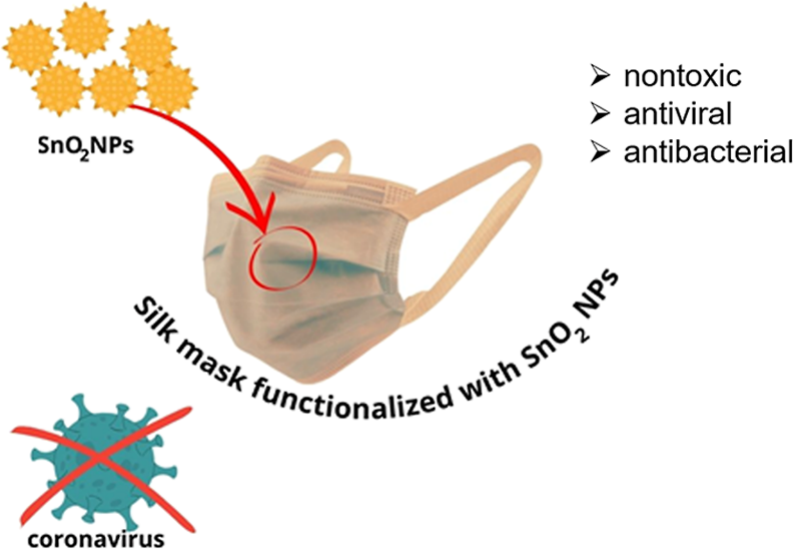

The COVID-19 pandemic has created a need to develop protective textiles that reduce the infection of SARS-CoV-2, mainly via face masks. The key issue in designing protective textiles is the functionalization with antiviral agents. This report presents tin oxide nanoparticles (SnO_2_NPs) as a novel, efficient antiviral agent against human coronavirus HCoV 229E due to blocking virus entry, attachment, and penetration into MRC-5 cells and nontoxicity. SnO_2_NPs were obtained by sodium stannate hydrolysis and have a 3 nm diameter, a cubic structure, and a zeta potential of −28.8. SnO_2_NPs were applied to functionalize a protective face mask made of silk fibroin. Polydopamine was applied to immobilize the particles. SnO_2_NPs have a negative potential and enhance silk fabric hydrophobicity, which is crucial for antiviral properties. The mask functionalized with SnO_2_NPs reveals very good antiviral properties and antibacterial activity against Gram-positive and -negative bacteria. Silk fabric functionalized with SnO_2_NPs retains the silk fibroin β-sheet structure, is nontoxic, noncorrosive to human skin, and reveals high thermophysiological wear comfort.The highest filtration efficiency is obtained for the 3-layered mask (60%), while breathing resistance, sufficient for the FFP3 mask, was achieved for the 1-layered mask (maximum allowable breathing of 100 and 300 Pa, respectively, for 30 L/min and 95 L/min inhale and 300 Pa for an exhale flow rate of 160 L/min). SnO_2_NPs can be useful in developing advanced antiviral textile materials to control virus spread and future pandemics.

## 1 Introduction

Since the outbreak of the coronavirus disease of COVID-19, the world’s attention has intensified on designing surfaces for inhibiting the spread of viral pathogens. Protective textiles, such as masks or clothes, functionalized with antimicrobial agents play a crucial role in mitigating or avoiding the spread of microorganisms.^1,2^ The most commonly applied textiles for designing protective masks are PP (polypropylene), PET (polyester), and sustainable materials such as natural silk.^3^

Various household textile materials were studied for potential applications in personal protective equipment, and only silk fabric was selected as a convenient candidate for these purposes based on particle filtration performance and breathability.^4^

Silk fabric is suitable for designing protective, antiviral textiles, including protective masks. Silk fibers are made of the natural product silk fibroin protein, which forms the fabric fibers. Silk fibroin is highly biocompatible, is characterized by low toxicity, and does not cause inflammation. Silk fabric is highly breathable, extremely water-repellent, hydrophobic, and effectively impedes the permeation of liquids and aerosolized water droplets.^5^ Moreover, silk fabric is biodegradable, which is crucial in designing sustainable protective masks, which do not contain any hazardous molecules and do not cause environmental pollution. Silk fabric is biocompatible and nontoxic but needs functionalization with antiviral agents to be an efficient protective textile against coronaviruses. There are many approaches for the modification of textiles to make them antiviral.

The development of antiviral textiles for personal protective purposes has been categorized into metal-based, carbon-based, and polymer-based materials for their surface modification.^6^ Nanostructures are the key modifiers, also for textiles, to combat COVID-19 pandemic.^7^ A metal-free quaternary ammonium-based coating was developed for hospital curtains, showing excellent antibacterial activity against various pathogens and >99% viral reduction with murine hepatitis virus (MHV).^8^ Additionally, textiles modified with copper (Cu) through magnetron sputtering exhibited strong antibacterial activity against *Staphylococcus aureus* and *Klebsiella pneumoniae*, along with good antiviral activity against vaccinia virus (VACV), herpes simplex virus type 1 (HSV-1), and influenza A virus H1N1 (IFV).^9^ The chemical modification of cellulose with poly((2-dimethylamino) ethyl acrylate) methyl chloride quaternary salt resulted in a significantly reduced viral activity by specifically functionalized cellulose.^10^

Due to the COVID-19 pandemic, materials with antiviral properties, including metals,^11^ polymers, and biopolymers, have been studied extensively to find efficient materials for mitigating the pandemic.^12^ Polymers with antiviral properties, such as those containing metal ions or electrically charged moieties, have been explored for on-contact virus elimination.^13^ Metal oxide structures have demonstrated antiviral characteristics; for example, ZnO therapods reveal activity against herpes simplex virus type 2 (HSV-2).^14^ Cupric oxide (CuO) can rapidly reduce infection by SARS-CoV-2.^15^ Silver has also been reported as an antiviral agent,^16^ for example, against the influenza A virus.^17^ However, SnO_2_ has not been reported previously as an antiviral material; SnO_2_ nanoparticles synthesized using laser ablation in water have shown antibacterial activity against *S. aureus* and *Escherichia coli.*(18) Moreover, SnO_2_ exhibited potent anticancer properties against esophagus squamous carcinoma cells.^19^ Despite the extensive experimental research carried out recently on SnO_2_ properties and applications,^17,20^ its antiviral properties have not been reported so far.

In this report, SnO_2_NPs were synthesized and characterized, and their antiviral potential was shown experimentally on MRC-5 cells for the first time. The antibacterial properties of SnO_2_ were confirmed against Gram-negative and -positive bacteria. It was shown how SnO_2_NPs modulate the influence of human coronavirus HCoV 229E virus on the cells: entry, attachment, and penetration into the cells. The face-protective mask was designed using a biodegradable and nontoxic material of silk fabric and a not-so-far-used antiviral agent of SnO_2_NPs. The antibacterial properties of SnO_2_NPs were confirmed on silk fabric against Gram-negative and -positive bacteria. The cytotoxicity tests revealed no toxic influence of the particles and functionalized silk fabric, which is also not corrosive on human skin. The thermophysiological comfort, surface wettability, filtration efficiency, and breathing resistance of the designed mask were studied to examine its further usefulness.

## 2 Experimental Section

### 2.1 Synthesis of SnO_2_ Nanoparticles

Aqueous solution of sodium stannate trihydrate Na_2_SnO_3_·3H_2_O (Merck) was prepared at a concentration of 0.25 wt %. 10 ml of sodium stannate trihydrate solution was added to 40 mL of boiling water, heated at 100 °C under reflux, and stirred at 600 rpm for 15 min. The sample was centrifuged four times at 18,000 rpm for 20 min and redispersed in deionized water. After the last washing, a sediment of SnO_2_NPs was redispersed in 50 mL of deionized water.

### 2.2 Functionalization of Silk Fabric with SnO_2_ Nanoparticles

Pure woven silk fabric with a plain wave used in this study has a thickness of 0.2 mm, a linear mass of weft and warp yarns of 37 dtex, and a mass per unit area of 70 g/m^2^. The functionalization of silk fabric with SnO_2_NPs was a two-step process. In the first step, silk fabric was covered with polydopamine (PDA) in the direct polymerization of PDA from dopamine hydrochloride on the fabric (silk_PDA). The pieces of silk fabric (15 cm × 20 cm) were immersed and stirred (600 rpm) in 1 L of an aqueous solution of dopamine hydrochloride (2-(3,4-dihydroxyphenyl)ethylamine hydrochloride 2%wt., Fluorochem) for 24 h. To obtain the alkaline pH essential for the polymerization process, Tris/glycine buffer (25 mM Tris and 192 mM glycine, pH 8.3, Bio-Rad) 1:10 (v/v) was added to the solution before immersion of the sample. After the polymerization, the samples were rinsed in deionized water three times.

The second step of the functionalization was related to the deposition of SnO_2_NPs on silk_PDA (silk_PDA_SnO_2_NPs). The silk_PDA fabric was dip-coated in aqueous SnO_2_NP colloid. It was dried and dip-coated again. This process was repeated three times. The samples were also rinsed three times after the second functionalization step to remove unbound particles from the silk surface.

### 2.3 Characterization of SnO_2_NPs and Silk Fabric Functionalized with SnO_2_NPs

#### 2.3.1 Scanning Electron Microscopy and Energy Dispersive X-ray Spectroscopy Studies

Scanning electron microscopy (SEM) was used to analyze the surface of the silk fabric before and after functionalization. VEGA 3 TESCAN SEM microscope was used to visualize the sample’s surface at the accelerating voltage of 20 kV. Prior to the measurements, the samples were covered with a 3 nm thick layer of gold. The elemental composition of the samples before and after the functionalization was studied using EDX (X-ray microanalyzer INCA Energy, Oxford Instrument Analytical) from five different 4.67 mm^2^ areas of each sample, with an accelerating voltage of 20 kV and a pressure of 20 Pa.

#### 2.3.2 Transmission Electron Microscopy and Inductively Coupled Plasma Mass Spectrometry Studies

Transmission electron microscopy (TEM) investigations were conducted using a Talos F200X transmission microscope at 200 kV. The measurements were performed in TEM and STEM modes using the high-angle annular dark-field (HAADF) detector. EDX was used for chemical composition mapping, which allowed us to obtain EDX maps of a single chemical element of the particles. The specimens for TEM investigations were suspended in ethanol and dropped onto amorphous thin carbon embedded on a Cu grid (300 Mesh Cu Pacific Grid Tech).

Inductively coupled plasma mass spectrometry (ICP–MS, Agilent 7900) was used to quantify Sn deposited on silk fabric.

#### 2.3.3 Fourier Transform Infrared Spectroscopy Spectral Analysis

An FTIR spectrometer (BRUKER Vertex 70 with a diamond ATR) was used for silk fabric characterization in a spectral range from 600 cm^–1^ to 4000 cm^–1^, with a resolution of 4 cm^–1^.

#### 2.3.4 Surface Wettability and Zeta Potential Determination

The wettability of the samples was measured using a goniometer PGX (Fibro System AB). Water was automatically applied with the goniometer at a constant volume of 4 μL (±0.2 μL) to the tested fabric surface. At least ten measurements were taken for each sample to calculate the average water contact angle.

The zeta potential of SnO_2_NPs was evaluated at 25 °C using a Zetasizer Nano ZS, Malvern Instruments.

#### 2.3.5 Thermogravimetric Studies

TGA (TG 209F1 Libra, Netzsch) measurements were performed at least three times for each sample. The weight of the samples was about 4 mg; they were placed in a ceramic crucible of 85 μL volume. The thermal measurements were carried out in the temperature range of 20 to 800 °C. The nitrogen flow was 25 mL/min, and temperature increased by 10 °C/min.

#### 2.3.6 Thermophysiological Comfort Studies

To study the thermophysiological comfort of the fabric on the skin, water vapor resistance and relative water vapor permeation were determined using the Permetest (Sensora) device. A fast response measuring instrument (working principle of Skin Model) allows the nondestructive determination of water-vapor permeability of fabric according to the PN-EN ISO 11092:2014–11 standard “Textiles. Physiological effects. Measurements of thermal and water-vapor resistance under steady-state conditions (sweating guarded hot-plate test)”. The thermal conductivity, diffusivity, and absorption were determined using the Alambeta (Sensora) device.

#### 2.3.7 Antiviral Property Determination

The antiviral properties were studied according to the ISO 18184:2019 standard “Textiles—Determination of antiviral activity of textile products”, using human coronavirus strain HCoV 229E (ATCC VR-740) and human lung fibroblasts cell line MRC-5 (ATCC CCL-171). The cells were cultured in minimum essential medium Eagle (MEM) media and 2% fetal bovine serum (FBS). The virus suspension was deposited on fabric samples (2 × 2 cm) for 2 h, without interfering substances, at a temperature of 22 ± 2 °C. The procedure was finished by adding 20 mL of cell culture medium. Virus titers were calculated as TCID/mL according to the Spearman-Karber’s method. ISO 18184:2019 standard defines that reduction of the virus titers equal or higher than 2 logs means good antiviral activity, while equal or higher than 3 means very good antiviral activity.

Three tests called as preincubation, attachment, and penetration were carried out to study the interaction of the virus with SnO_2_NPs. They were performed on the MRC-5 (ATCC CCL-171) cell line and human coronavirus strain HCoV 229E (ATCC VR-740). MRC-5 cells were cultured in 24-well plates.

In the preincubation assay, the virus and SnO_2_NP colloid were incubated together at room temperature for 1 h before being applied to the cell culture. Following an hour of adsorption, the medium was replaced with a fresh medium to continue the experiment.

To study the impact of the colloid on viral attachment, cells were prechilled at 4 °C for 15 min and then cotreated with the SnO_2_NP colloid and HCoV 229E for 1 h at 4 °C. Subsequently, the inoculum was removed, cell monolayers were washed with ice-cold PBS, and the cultures were incubated at 37 °C. After 24 h post-infection, viral titers were quantified using qPCR.

The viral penetration assay was initiated by prechilling cells at 4 °C for 15 min, followed by infection at 4 °C for 1 h to facilitate viral binding without entry. After removing the inoculum, cells were washed with ice-cold PBS, and the SnO_2_NP colloid was applied for 2 h at 37 °C. The colloid was then removed, cells were washed twice with cold PBS, and the incubation was continued for an additional 18 h at 37 °C. Viral titers were determined via qPCR.

RNA extracted from each sample was analyzed using Real-Time RT-qPCR with the QuantiNova RT-qPCR Kit (Qiagen Inc., Santa Clarita, CA, USA) on a QuantStudio 5 thermocycler (Applied Biosystems, Foster City, CA, USA). The RT-qPCR protocol was conducted following the manufacturer’s guidelines. A total of 2 μL of extracted RNA (500 ng total concentration) was combined with 10 μL of Probe RT-PCR MasterMix, 6 μL of RNase-free water, 1 μL of Probe specific for HCoV 229E, and 0.2 μL of QN Probe RT-Mix, adjusted with water to a final reaction volume of 20 μL. The cycling conditions were as follows: reverse transcription (RT) at 45 °C for 10 min, initial PCR heat activation at 95 °C for 5 min, and 40 cycles of a two-step process: denaturation at 95 °C for 5 s, followed by annealing and extension at 60 °C for 30 s. All reactions were performed in triplicate to ensure reproducibility. The standards for PCR quantification were prepared using RNA extracted from HCoV-infected cells with a TCID_50_ value of 5/mL. The extracted RNA was serially diluted to obtain a standard curve for qPCR analysis.

#### 2.3.8 Antibacterial Property Determination

The antibacterial properties of SnO_2_NPs were studied according to the AATCC Test Method 100–2012 “Antibacterial Finishes on Textile Materials: Assessment of” standard against Gram-positive bacteria *S. aureus* (ATCC 6538) and Gram-negative *Klebsiella pneumonie* (ATCC 4352). Inoculum concentration for both bacteria strains was about 2.6 × 10^5^ CFU/mL. The determination of bacterial growth reduction was evaluated based on the PN-EN ISO 20743 standard “Determination of the antibacterial activity of textile products”. The bacterial growth was evaluated after 21 to 24 h incubation at 37 ± 2 °C. The bacteriostatic and biocidal coefficients were calculated based on the JIS L 1902 standard “Testing for antibacterial activity and efficacy on textile products”.

#### 2.3.9 Cytotoxicity and Skin Corrosion Studies

The cytotoxicity of SnO_2_NPs and silk fabric functionalized with SnO_2_NPs was determined using the WST-1 test. Two cell lines were tested: human lung carcinoma: A549 (ATCC CCl-185) and human keratinocytes: HaCaT (CLS Cell Lines Service GmbH). The cell culture medium was Dulbecco’s modified Eagle′s medium (DMEM) medium and 10% FBS. Extract from textile structures was prepared according to the ISO standard 18,184:2019—“Textile—Determination of the antiviral activity of textile products”. The undiluted extract (dil0) and diluted (diluted 1:1, diluted 1:5) were added to the cells for 24 h at 37 ± 0.5 °C. The cytotoxicity was evaluated spectrophotometrically, and the absorbance intensity of formazan at 440 nm was evaluated. The material was considered toxic if the cell viability of undiluted sample was below 50% of viable cells and weakly toxic if the cell viability of undiluted samples was between 50 and 80%. The viability of 80% of cells treated with undiluted samples was considered the highest concentration showing no toxicity.

The skin corrosion induced by silk fabric functionalized with SnO_2_NPs was analyzed according to OECD test guideline 431 Skin Corrosion. The test was performed on a 3D skin model EpiSkinTM, consisting an in vitro reconstructed human epidermis from normal human keratinocytes and dermis from normal human fibroblasts. The skin model was exposed to pure silk fabric and silk fabric functionalized with SnO_2_NPs for 60 min at 37 ± 0.5 °C. Then, inserts with the skin model were rinsed 15 times with PBS. Next, the skin models were further incubated for 24 h. The toxicity was determined by the evaluation of the absorbance intensity of formazan salt after 3 h of incubation (WST-1 test). According to the classification of EU and Globally Harmonized System of Classification and Labeling of Chemicals (GHS, R38/category 2), a substance is irritant when the viability of three single models exposed to it is below 50% of the average viability of the negative control.

#### 2.3.10 Mask Filtration Efficiency and Breathing Resistance

The filtration efficiency of functionalized silk fabric was measured using an automated filter tester (GT-RA09B-1, Gester Instruments Co., Ltd.). The air flow was 30 L/min, and measurement time was 30 s. The particle aerosol for evaluation filtration efficiency was formed from 5%wt. NaCl aqueous solutions. The particles were neutralized, and their median diameter was 0.075 ± 0.02 μm. The measurements were conducted at a temperature of 23 ± 1 °C and relative humidity of 42 ± 1%.

Inhalation and exhalation resistance of silk fabric functionalized with SnO_2_NPs were measured by a GT-RA03 Mask and Respirator Breathing Resistance Tester (Quanzhou Gester International Co., Ltd.). The inhalation resistance was measured at the flow rate of 30 and 95 L/min while the exhalation resistance at 160 L/min. The breathing resistance was evaluated according to EN 149:2001 + A1:2009 standard “Respiratory protective devices—Filtering half masks to protect against particles—Requirements, testing, marking”. The measurements were conducted at 22 ± 1 °C and relative humidity at 56 ± 2%.

#### 2.3.11 Statistical Method

Data were analyzed using Student’s *t*-test.

## 3 Results and Discussion

### 3.1 Structure, Morphology, and Properties of SnO_2_NPs and Silk Fabric Functionalized with SnO_2_NPs

**Figure 1:**
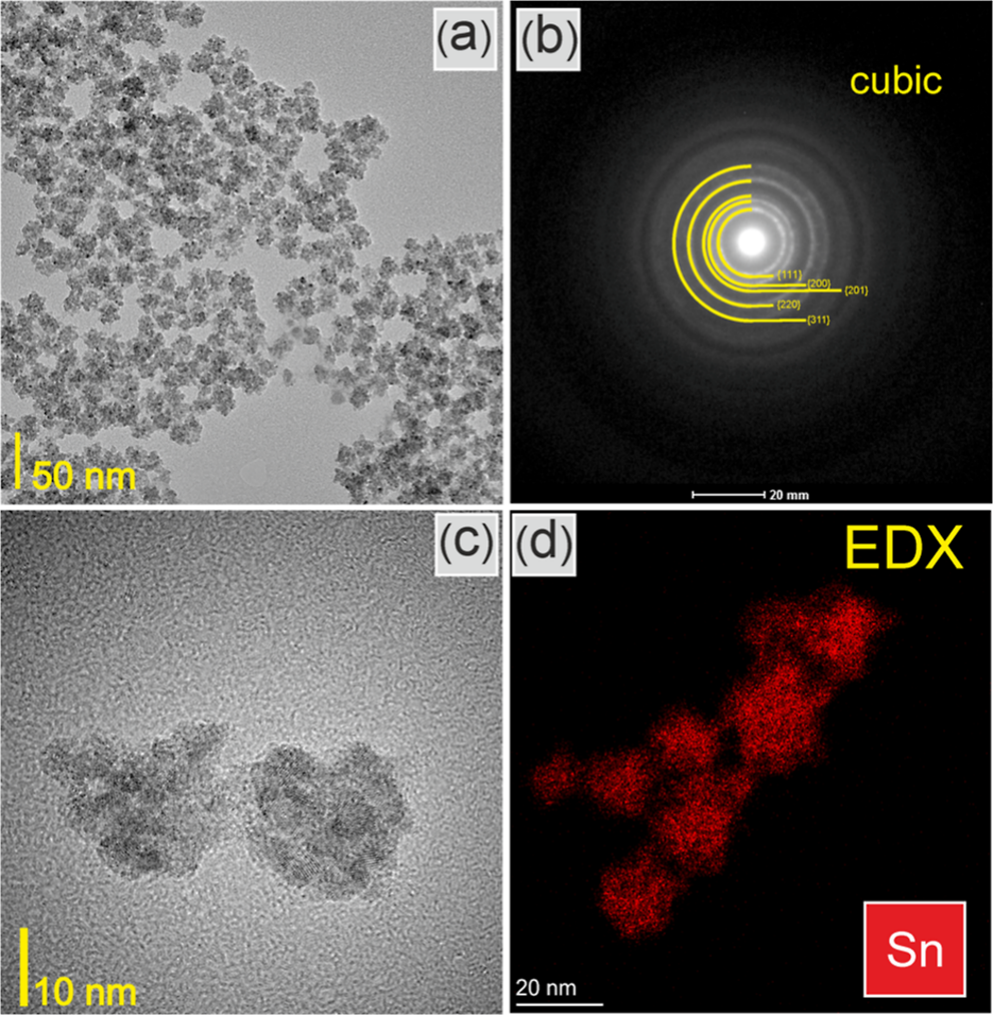
(a,c) TEM images, (b) electron diffraction pattern, and (d) Sn EDX map of SnO_2_NPs.

Silk fabric was functionalized with SnO_2_NPs to make it an efficient protective material. Silk fabric was selected due to its high biocompatibility, water-repellent ability, and water droplet-passing protection.^3^ To the best of our knowledge, this is the first report on the antiviral properties of SnO_2_NPs. In this report, SnO_2_NPs were synthesized in a hydrolysis reaction of sodium stannate to the formation of Sn(OH)_6_^2–^ions (reactions 1–3) and then SnO_2_ (eq 4) due to high temperature.^21^
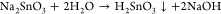
1
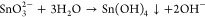
2
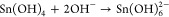
3
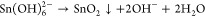
4In our previous report, sodium stannate hydrolysis was applied to form a SnO_2_ protective shell on silver nanoparticles (AgNPs).^21^ Sodium stannate hydrolysis in the presence of silver nanowires (AgNWs) also formed a SnO_2_ shell on their surface.^22^ The shell was made of 7 nm rutile-type crystals, the most typical SnO_2_ crystal structure. In this report, SnO_2_ was not formed in the presence of other nanostructures; it was a simple hydrolysis of sodium stannate in water, which caused the formation of smaller crystals of about 3 nm, and their structure was cubic (Figure 1). The synthesis of cubic SnO_2_ crystals has been previously successfully demonstrated through different approaches, showcasing the feasibility of obtaining this crystal structure. Yu et al. synthesized nanostructured cubic SnO_2_ crystals through a solvothermal route, with particles being rutile structured and almost uniformly cube-shaped in quantum size.^23^ Another investigation reported the synthesis of Al-doped SnO_2_ nanowires, where Al-induced a change in SnO_2_ crystals from a tetragonal rutile to a cubic structure.^24^ Obtaining cubic SnO_2_ is feasible based on the diverse synthesis approaches and structural modifications explored in the literature.

TEM images (Figure 1a,c) show 10 to 20 nm agglomerates composed of 3 nm single crystals of SnO_2_. The electron diffraction pattern (Figure 1b) indicates the cubic structure of SnO_2_ nanoparticles with no evidence of any impurity phase. The EDX map (Figure 1d) reveals the presence of tin in the particles. The crystals tend to agglomerate and form polycrystalline agglomerates of several nanometers.

**Figure 2:**
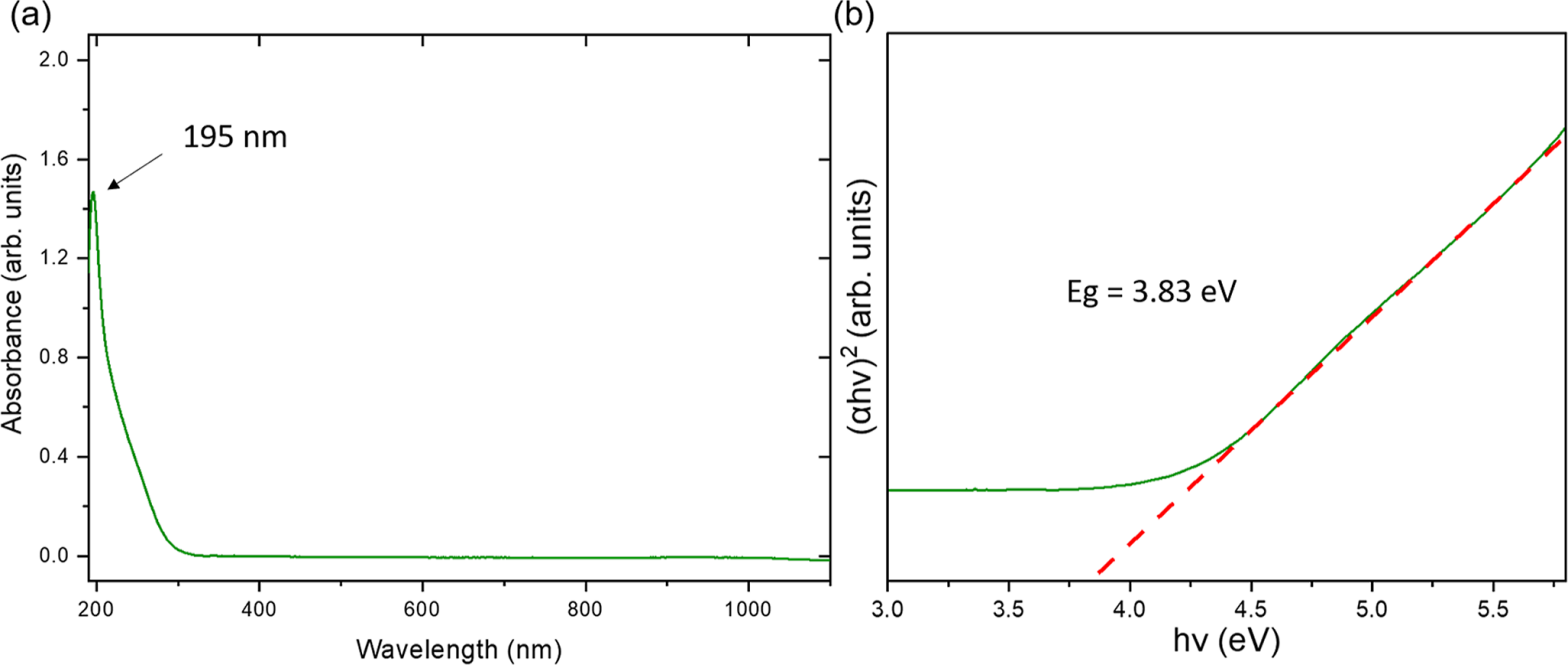
(a) Absorbance spectra of SnO_2_NPs and (b) Tauc plot for SnO_2_ energy gap value determination.

The absorbance spectrum of the SnO_2_NP colloid (stock/working solution) reveals the band with the maximum at 195 nm (Figure 2a). The value of the band gap for SnO_2_NPs, which are semiconducting nanostructures, was calculated based on the Tauc plot (Figure 2b) to be about 3.83 eV, a typical value for tin oxide nanoparticles.^25^

### 3.2 Characterization of Silk Fabric Functionalized with SnO_2_NPs

**Figure 3:**
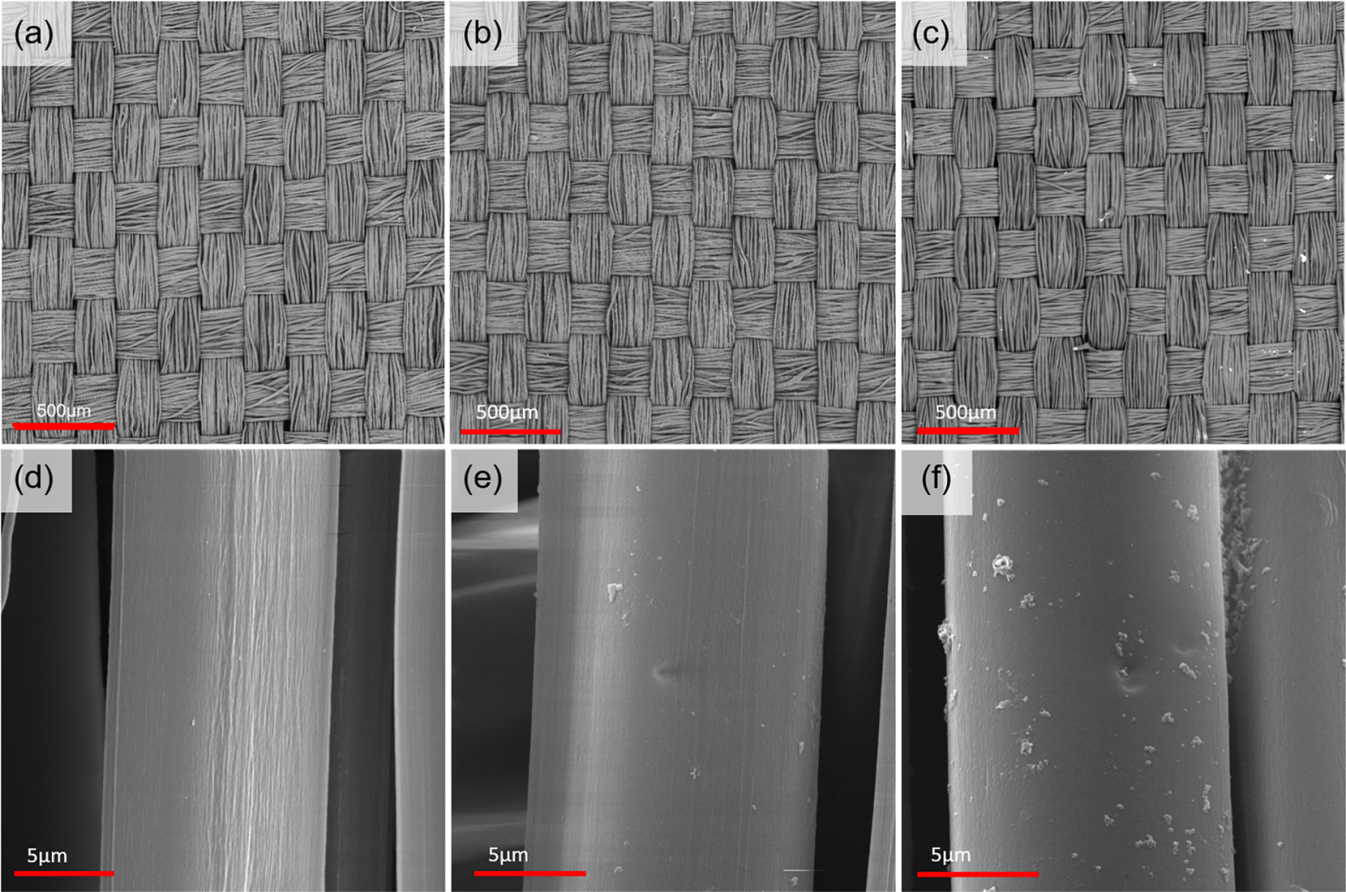
SEM images of (a,d) silk fabric, (b,e) silk fabric functionalized with PDA, and (c,f) silk fabric functionalized with PDA_SnO_2_NPs.

The aim of this study was to analyze SnO_2_NPs as an antiviral agent and design a material for a face mask functionalized with SnO_2_NPs to evaluate their properties as a part of a protective textile structure. Silk woven fabric as a model textile was functionalized with SnO_2_NPs using polydopamine as a linker between the fabric surface and the nanoparticles. The process of polymerization was carried out directly on the fabric. Figure 3 shows SEM images of silk fabric before (Figure 3a,d) and after PDA (Figure 3b,e) and PDA_SnO_2_NP functionalization (Figure 3c,f). The single SnO_2_NPs were not visualized on the silk fabric surface due to their small size of about 3 nm; only a few agglomerates of the particles are noted. The yarns of warp and weft remain unchanged after the functionalization process, but the structure is slightly loosened. The elemental composition of functionalized SnO_2_NP fabric analyzed by EDX was carbon about 49% wt., nitrogen about 21% wt., oxygen 31% wt., sulfur below 1% wt., and Sn below 1% wt. Table 1 summarizes the elemental composition of silk fabric, silk fabric with PDA, and silk fabric with PDA_SnO_2_NPs. Carbon, nitrogen, and oxygen are typical elements for silk fabric and PDA. The trace amount of sulfur indicates the natural origin of the silk due to the presence of a single disulfide bond to link heavy and light chains together in the protein structure.^26^ The presence of sodium in pure silk fabric indicates the residues of silk degumming agent sodium carbonate,^27^ which is completely rinsed in the functionalization process and was not detected for PDA and PDA_SnO_2_NP functionalized fabric. Tin presence indicates the functionalization of silk fabric with SnO_2_NPs.

**Table 1:** Results of EDX Analysis of Silk Fabric, Silk Fabric Functionalized with PDA, and Silk Fabric Functionalized with PDA_SnO_2_NPs

	C (%wt.)	N (%wt.)	O (%wt.)	S (%wt.)	Na (%wt.)	Sn (%wt.)
silk fabric	48.12 ± 0.14	20.97 ± 0.22	30.51 ± 0.14	0.08 ± 0.01	0.32 ± 0.01	
silk fabric with PDA	48.39 ± 0.03	20.94 ± 0.16	30.60 ± 0.13	0.07 ± 0.01		
silk fabric with PDA_SnO_2_NPs	48.53 ± 0.13	20.60 ± 0.25	30.63 ± 0.15	0.07 ± 0.01		0.18 ± 0.03

**Figure 4:**
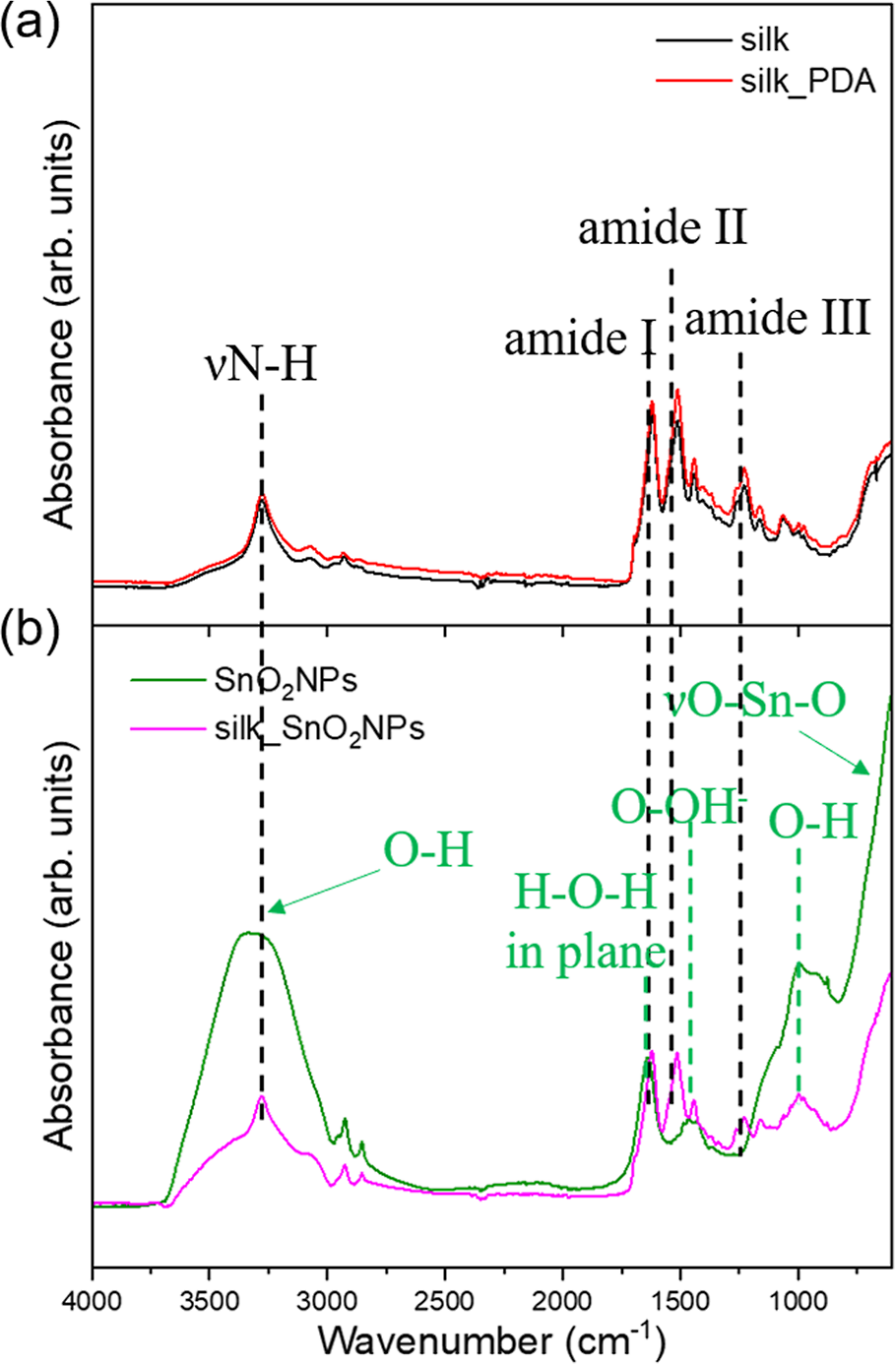
IR spectra of (a) silk fabric and silk fabric functionalized with PDA; (b) SnO_2_NPs and silk fabric functionalized with PDA and SnO_2_NPs (silk_SnO_2_NPs).

IR spectroscopy allowed the study of functional groups and chemical bonds of the silk fabric before and after functionalization (Figure 4). The main peaks characteristic of silk fibroin were noted at 1618, 1512, and 1262 cm^–1^, indicating β-sheet crystallites of amide I, II, and III, respectively (Figure 4a). They were present and not shifted after the functionalization with PDA and further with SnO_2_, indicating that the β-sheet structure of silk fibroin was not affected by the functionalization process.

The spectrum for SnO_2_ powder (SnO_2_NPs after the synthesis) reveals an intense, broad band around 500–800 cm^–1^ ascribed to Sn–O stretching vibration (Figure 4b).^28^ The broad band at around 3325 cm^–1^ is attributed to the O–H stretching,^28^ and the bands at 1456 and 988 cm^–1^ are assigned to the –OH groups attached directly to the surface oxygen of SnO_2–*x*_.^29^ The absorption band at 1640 cm^–1^ is related to H–O–H in-plane deformation.^29^ Described above, bands typical for SnO_2_ structures also appeared in the spectra of silk fabric functionalized with the particles, indicating SnO_2_NP’s presence on fabric and efficient process of functionalization.

**Figure 5:**
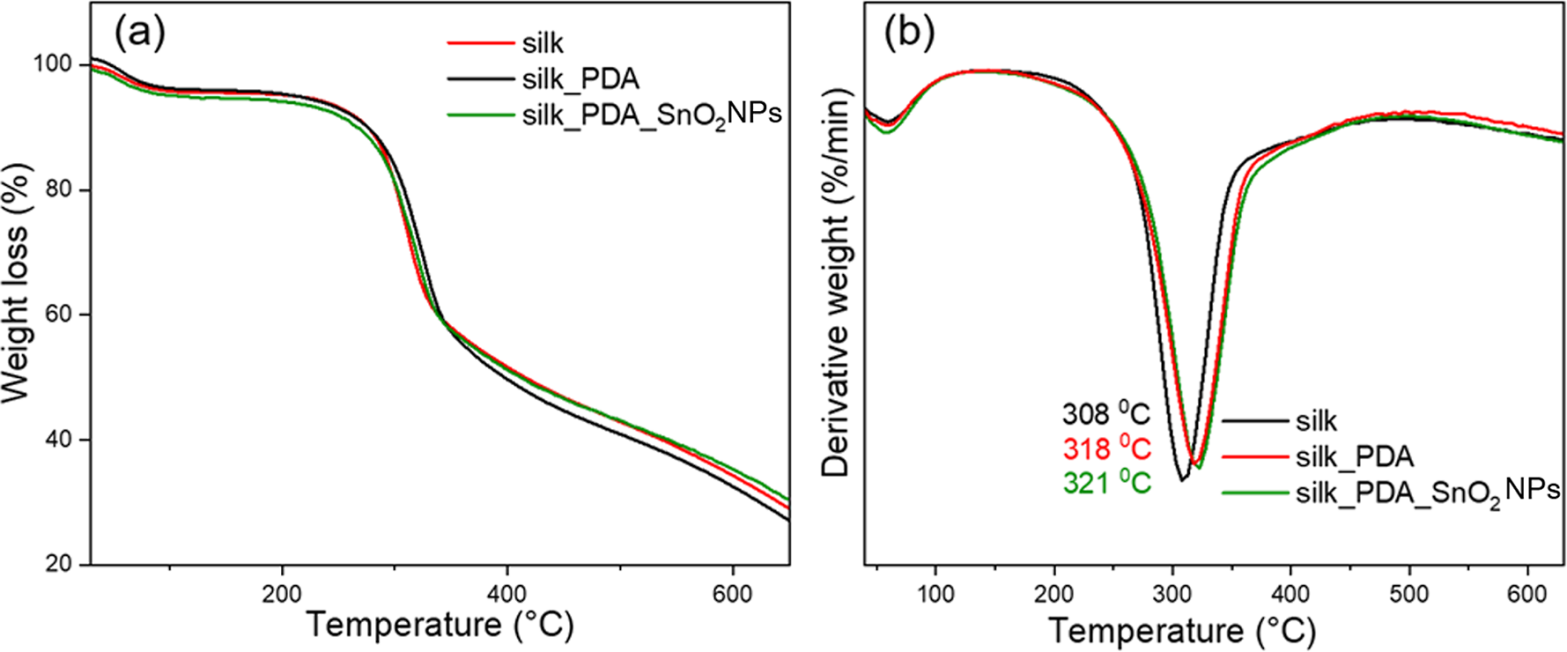
(a) TG and (b) DTG thermograms of silk fabric and silk fabric functionalized with PDA (silk_PDA) and with PDA and SnO_2_NPs (silk_PDA_SnO_2_NPs).

The thermal properties of the silk fabric slightly changed after the functionalization process (Figure 5) due to the deposition of PDA and further SnO_2_NPs on the fabric surface. The weight loss during the thermal decomposition of the silk fabric indicates below 1% wt. SnO_2_NPs. The thermal decomposition peak after the functionalization slightly shifted to higher temperatures, from 308 °C typical for silk-based materials^27^ to 318 and 321 °C, respectively, for silk modified with PDA and PDA_SnO_2_NPs. It was found in our previous reports that the functionalization of silk fabric with PDA improved the thermal properties of silk fabric, and the main thermal decomposition process occurs at temperatures a few degrees Celsius higher.^30,31^ The SnO_2_NPs can additionally shift the process to higher temperatures due to the high thermal stability of SnO_2_. Its thermal decomposition occurs in the temperature range of 1300 to 1500 °C.^32^ Since the amount of particles introduced is small, they cannot fully thermally protect the silk fabric. It is also not necessary for antimicrobial applications and the design of protective masks; in fact, a smaller number of particles is preferred for lower cytotoxicity and avoiding negative effects on the skin.

To determine the exact amount of tin on the fabric, ICP–MS technique was applied. It was found that there is 155 ± 30 mg of Sn per kilogram of the fabric. To the best of our knowledge, there is no report on antiviral textile structure functionalization with SnO_2_NPs to compare the degree of nanoparticle deposition on the fabric. Zhang et al. found that for silk fabric functionalized with silver nanoparticles, increasing the silver content from 98.65 to 148.68 mg/kg has no significant change in antibacterial properties.^33^ In the case of cotton fabric functionalization with AgNPs, the Ag content of more than 180 mg/kg caused a significant reduction of bacteria.^34^ ZnO and Ag nanoparticles are known as efficient antimicrobial agents. SnO_2_NPs with a similar degree of deposition on the textile structure when demonstrating antibacterial and antiviral properties make SnO_2_NPs a highly competitive material.

**Figure 6:**
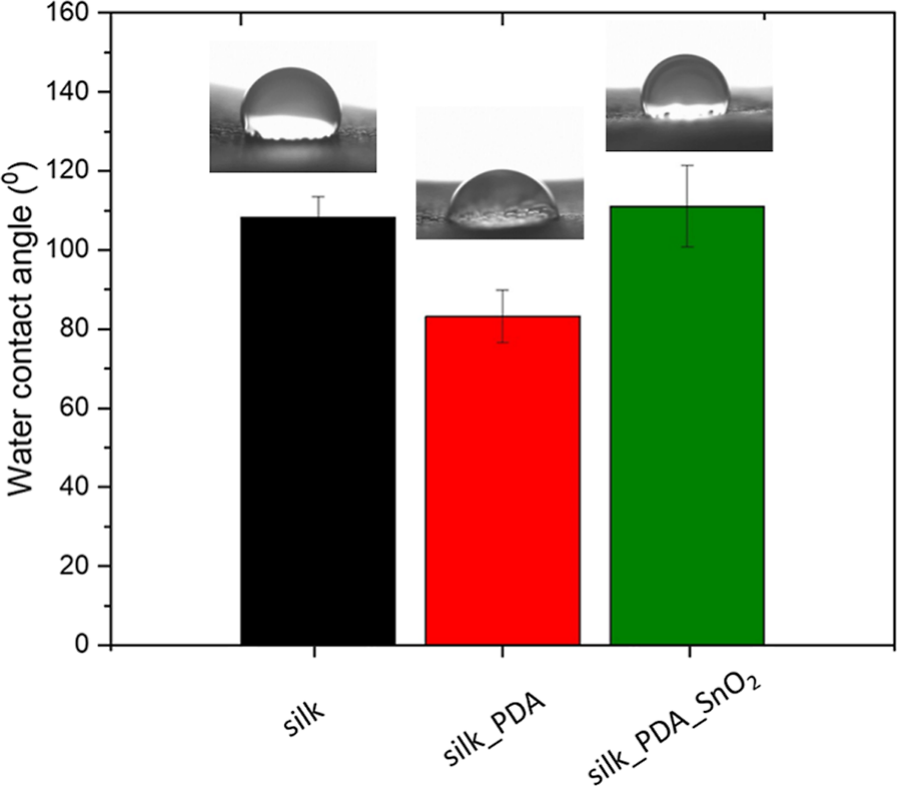
Water contact angle of silk fabric and silk fabric functionalized with PDA and with PDA_SnO_2_NPs.

The water contact angles of silk fabric, silk with PDA, and PDA_SnO_2_NPs are, respectively, 108 ± 5°, 83 ± 6°, and 111 ± 10° (Figure 6). Silk fabric is hydrophobic because it is made of silk fibroin protein, mainly composed of two hydrophobic amino acids, glycine (43%) and alanine (30%). Silk fibroin fibers form in the cocoon the hydrophobic membranes, which protect the growing moth against harsh abiotic and biotic conditions. It was also shown that silk is a hydrophobic barrier to droplets.^5^ After functionalization with PDA, silk fabric becomes hydrophilic due to PDA’s high hydrophilicity created by polar functional groups.^35^ It is a well-known phenomenon that PDA coating makes various surfaces hydrophilic.^36^ The value of the water contact angle of silk_PDA confirms the formation of a PDA layer on silk fabric. SnO_2_NP functionalization makes silk_PDA fabric hydrophobic, which is crucial for designing antiviral textiles. SnO_2_ is insoluble in water and characterized by hydrophobic nature. The water contact angle is slightly greater than that of pure silk fabric; it is probably also related to the structure of SnO_2_NP aggregates, which trap air in the interspaces between single tin oxide crystals. It was reported by Chen et al. that the rough surface of SnO_2_ revealed a higher water contact angle than the smooth one, and the SnO_2_ nanostructures with extended surface area and high surface-to-volume ratio can be superhydrophobic.^37^ Hydrophobic properties of protective textiles are crucial because water-based droplets stay longer on the surface and are not facile to absorb, while hydrophilic materials absorb droplets immediately.^5^ It was found that hydrophobicity or even superhydrophobicity is crucial in preventing fomite formation by repelling virus-laden droplets.^38^ A surface with lower adhesion offers better prevention of viral contamination. A superhydrophobic surface would be free of SARS-CoV-2 deposition when the adhesion is 1 mN; the fomite formation is completely suppressed for effectively preventing the fomite-to-human transmission of COVID-19.^38^

### 3.3 Antimicrobial Properties of Silk Fabric Functionalized with SnO_2_NPs

In recent years, SnO_2_ has gained attention mainly due to its antibacterial properties. Many reports have shown that SnO_2_NPs exhibit significant antibacterial activity against various bacteria.^39,40^Table 2 summarizes the reports on the antimicrobial activity of SnO_2_-based nanostructures, including this study. This report also evaluated the functionalized silk fabric for antibacterial properties against Gram-positive *S. aureus* and Gram-negative *K. pneumoniae* bacteria (Table 3). The reduction of bacterial growth of silk fabric functionalized with SnO_2_NPs was above 95% for both kinds of bacteria. The bacteriostatic coefficient (S) was calculated to be above 4.2 and 5.5 for selected Gram-positive and Gram-negative bacteria, respectively. The biocidal coefficient (L) was determined to be about 2.3 and 1.5 for Gram-positive and Gram-negative bacteria, respectively. According to the JIS L 1902 standard, textile structures exhibit bacteriostatic properties if the value of the S coefficient is not less than 2. The fabric reveals biocidal properties if the L coefficient is not less than 0. It indicates that silk fabric functionalized with SnO_2_NPs has excellent antibacterial properties, including high bacteriostatic and biocidal activity. Nonfunctionalized silk fabric does not reveal biocidal or even bacteriostatic activity. Silk_PDA fabric shows a slight bacteriostatic effect against both kinds of bacteria. The mechanism of antibacterial activity of metal oxide nanoparticles is related to the generation of reactive oxygen species (ROS)^40^ and the disruption of bacterial cell membranes. Some reports indicate the importance of the high surface-to-volume ratio of SnO_2_NPs.^39^ The large surface area of NPs enhances their contact with bacteria, further contributing to their antibacterial efficacy. The surface interactions between microbes and antimicrobial agents are crucial in antibacterial properties. Sn^4+^ ions can release and react with carboxy groups of the surface protein of bacteria cells, enhance the generation of ROS, and disrupt the integrity of the cell membrane.^41^

**Table 2:** Antimicrobial Activity of SnO_2_-Based Nanostructures

SnO_2_-based nanostructures	Gram-negative bacteria	Gram-positive bacteria	antiviral activity	references
SnO_2_NPs (solvotermal method)	E. coli			(39)
SnO_2_NPs (co-precipitation method)	S. flexineri	S. aureus		(40)
	E. coli			(40)
Cu/SnO_2_NPs	P. aeruginosa	S. aureus		(41)
SnO_2_ nanowires	E. coli	S. aureus		(47)
	K. terrigena	B. subtilis		(47)
		E. faecalis		(47)
		S. epidermidis		(47)
SnO_2_NPs (hydrolysis)	K. pneumoniae	S. aureus	HCoV 229E (human coronavirus)	present study

**Table 3:** Antibacterial Properties of Silk Fabric and Silk Fabric Functionalized with PDA and PDA with SnO_2_NPs against *S. aureus* and *K. pneumoniae*

	silk		silk_PDA		silk_PDA_SnO_2_NPs	
	S. aureus	K. pneumoniae	S. aureus	K. pneumoniae	S. aureus	K. pneumoniae
inoculum concentration CFU/mL	∼2.6 × 10^5^	∼2.6 × 10^5^	∼2.6 × 10^5^	∼2.6 × 10^5^	∼2.6 × 10^5^	∼2.6 × 10^5^
reduction of bacterial growth	0%	0%	21.93%	17.3%	>99.44%	>95.54%
bacteriostatic coefficient (S)	0.1	0.3	3.9	2.1	>4.2	5.5
biocidal coefficients (L)	–3.0	–4.2	0	–0.7	>2.3	1.5

**Figure 7:**
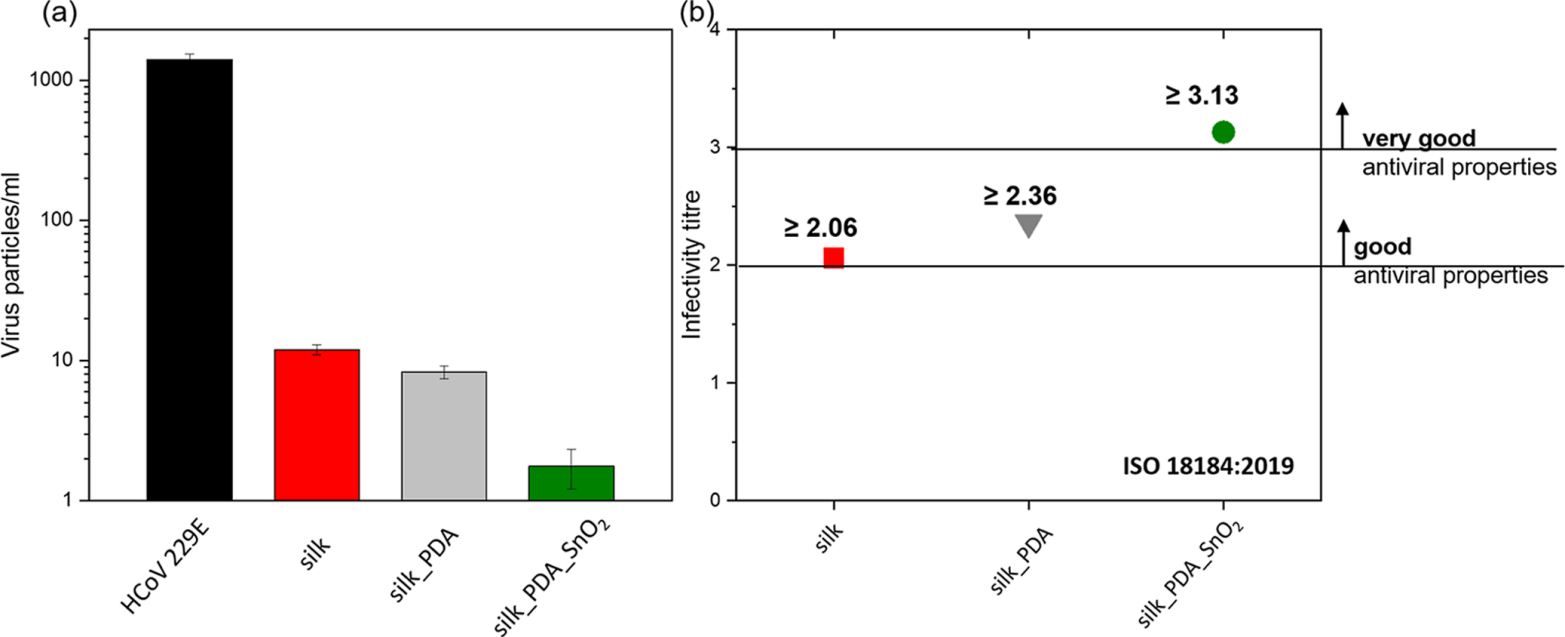
(a) Virus titers in medium without any treatment and after incubation with the silk fabric, silk fabric functionalized with PDA, and silk fabric functionalized with PDA and SnO_2_NPs. (b) Values of HCoV 229E titers for silk fabric, silk fabric functionalized with PDA, and silk fabric functionalized with PDA and SnO_2_NPs.

Although SnO_2_NPs have high antimicrobial potential, limited research has focused on their antiviral properties. Since the COVID-19 outbreak, some reports have appeared on coronavirus biosensors. Due to their unique surface properties, SnO_2_NPs are effective in detecting viruses. Their surface was functionalized with angiotensin II, which has a high affinity to spike proteins.^42^ In this report, SnO_2_NPs are presented as an antiviral agent to design a protective mask. The reduction of HCoV 229E virus on silk fabric and silk fabric functionalized with SnO_2_NPs is significant (Figure 7a). The infectivity titer, given as virus reduction of log 10 (TCID/mL), was calculated according to the ISO 18184:2019 standard to be ≥2.06, ≥2.36, and ≥3.15 for silk, silk functionalized with PDA, and silk functionalized with PDA and SnO_2_NPs, respectively (Figure 7b). The standard describes an infectivity titer and gives two significant values, first above log 2 and second above log 3, indicating good and very good antiviral properties, respectively. It indicates that pure silk fabric is a suitable material for designing antiviral textiles; it reveals good antiviral activity without functionalization, which is in agreement with other reports on silk fabric as a functional material for protective masks.^3,5^ PDA does not deteriorate the good antiviral activity of silk fabric. Silk fabric, after the functionalization with SnO_2_NPs, reveals very good antimicrobial properties, indicating SnO_2_NPs as an efficient agent against virus HCoV 229E.

**Figure 8:**
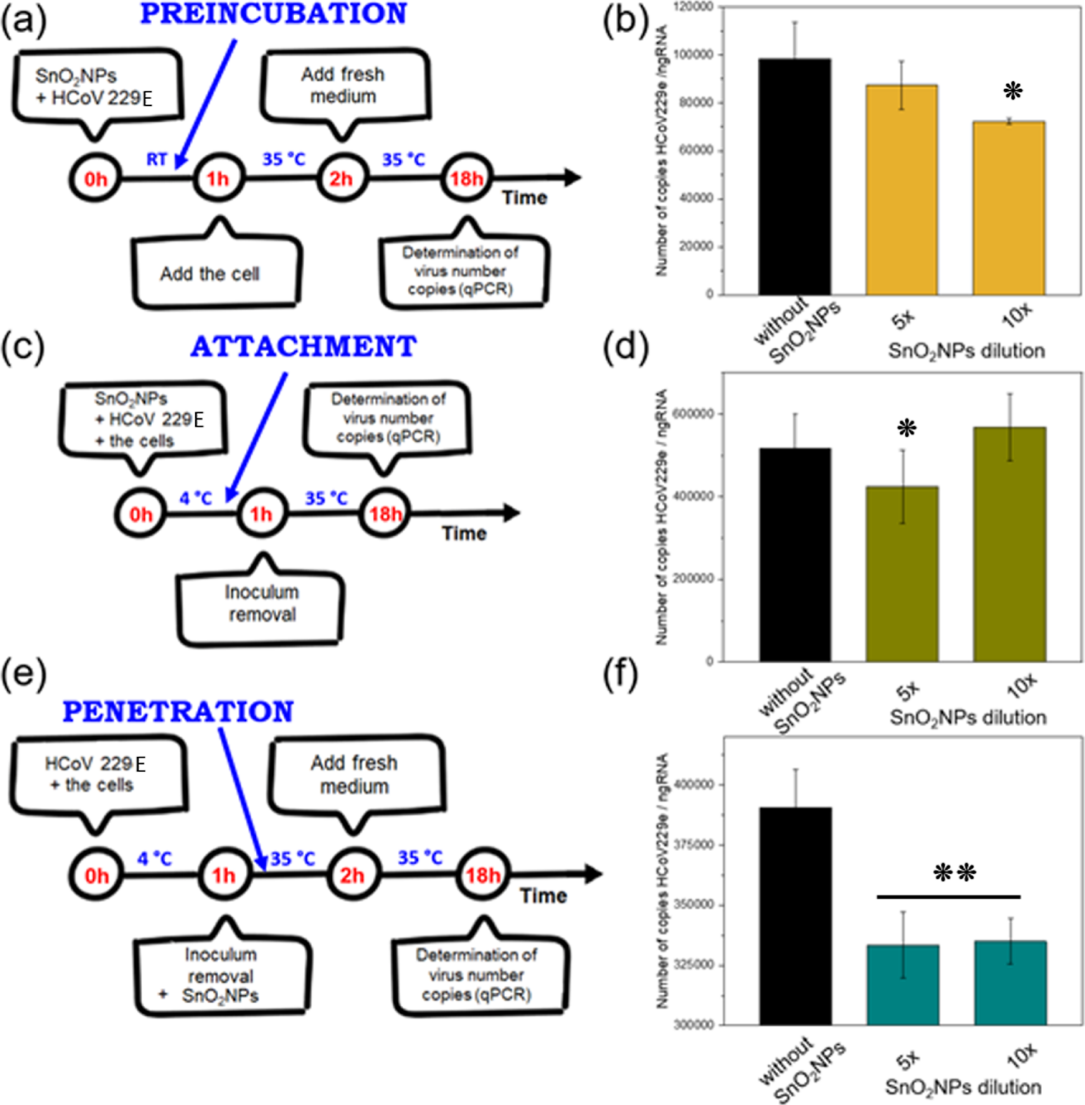
(a,c,e) Schemes of three experiments performed to analyze the influence of HCoV 229E virus treated with SnO_2_NPS on MRC5 cells and (b,d,f) their results (number of virus copies): (a,b) preincubation, (c,d) attachment, and (e,f) penetration. *represents significant differences with *p ≤ 0.05 and *p ≤ 0.001 in comparison to the control sample.

To study the influence of SnO_2_NPs on virus interaction with cells, three types of experiments were performed (Figure 8) called preincubation, attachment, and penetration. The first one, labeled as preincubation (Figure 8a), involves adding SnO_2_NPs to the virus solutions at room temperature, and only after 1 h, the cell culture is added. The incubation temperature was 35 °C, and the incubation total time was 18 h, although, after 2 h of incubation, a fresh medium was added. The experiments were performed for two different dilutions of SnO_2_NPs, the stock particle solution five and ten times diluted. The amount of viral RNA after the infection decreased for a higher concentration of the SnO_2_NPs, which is related to blocking virus entry to the cells by the particles (Figure 8b). The number of virus copies increased for both concentrations of SnO_2_NPs. The lower concentration of the particles had an even better effect, showing that even lower concentrations of the particles are efficient for blocking virus entry into the cell. Both concentrations (5× and 10× dilutions) are highly effective (Figure 8), and they can be used for antiviral purposes.

The second test is called attachment because it analyzes the ability of SnO_2_NPs to prevent the attachment of the virus to the cells (Figure 8c). The cells are inoculated with the virus at 4 °C for 1 h in the presence of SnO_2_NPs, then the inoculum is removed, and the cells were incubated for the next 17 h at 35 °C. SnO_2_NPs of higher concentration (5x dilutions) inhibit virus attachment to the cell (Figure 8d). The lower concentration of SnO_2_NPs is inefficient in inhibiting the virus attachment to the cells.

The third test is called penetration because its results show how SnO_2_NPs can influence virus penetration into the cell. The cells are inoculated with the virus at 4 °C for 1 h, then the inoculum is removed, and the particles are introduced; the cells were incubated for the next 17 h at 35 °C, but after 2 h of this incubation, a fresh medium was added (Figure 8e). The number of virus copies decreased significantly for both concentrations of SnO_2_NPs due to inhibiting the virus penetration into the cells (Figure 8f). The antiviral properties of SnO_2_NPs can be influenced by bond strength of the nanoparticles with the virus. The total structural charge of SARS-CoV-2 is positive.^43^ It was found that during the pandemic, the positive charge of the spike protein increased as the adaptation to human transmission.^44^ Zeta-potential of SnO_2_NPs was determined to be about −28.8 (±1.95) at pH 8.5. According to the literature, the zeta potential of SnO_2_ is negative for a pH value above 4;^45^ it is in the pH range for aqueous solutions of SnO_2_NPs obtained in this report.

The negatively charged SnO_2_NPs can easily interact with the virus and effectively harmfully influence the virus. It was found that negatively charged cupric oxide nanoparticle charge–charge interactions with positively charged virus envelope protein were the main reason behind the SARS-CoV-2 inactivation efficacy.^13^ Charge–charge interaction generated ROS and excitonic effects, which inactivate the virus.^46^

Moreover, a virus using a negative charge to interact with SnO2NPs lowers its bonding efficiency with cells and makes virus adaptation to cell transmission useless.

SnO_2_NPs have antiviral properties against HCoV 229E; they can inhibit virus entry, attachment, and penetration into the cells (MRC-5). Their action is dose-dependent; the most efficient antiviral properties are noted for only five times diluted stock particle solution. This concentration’s usefulness for biomedical purposes was also tested by evaluating its cytotoxic effect.

### 3.4 Cytotoxicity and In Vitro Skin Corrosion of Silk Fabric Functionalized with SnO_2_NPs

**Figure 9:**
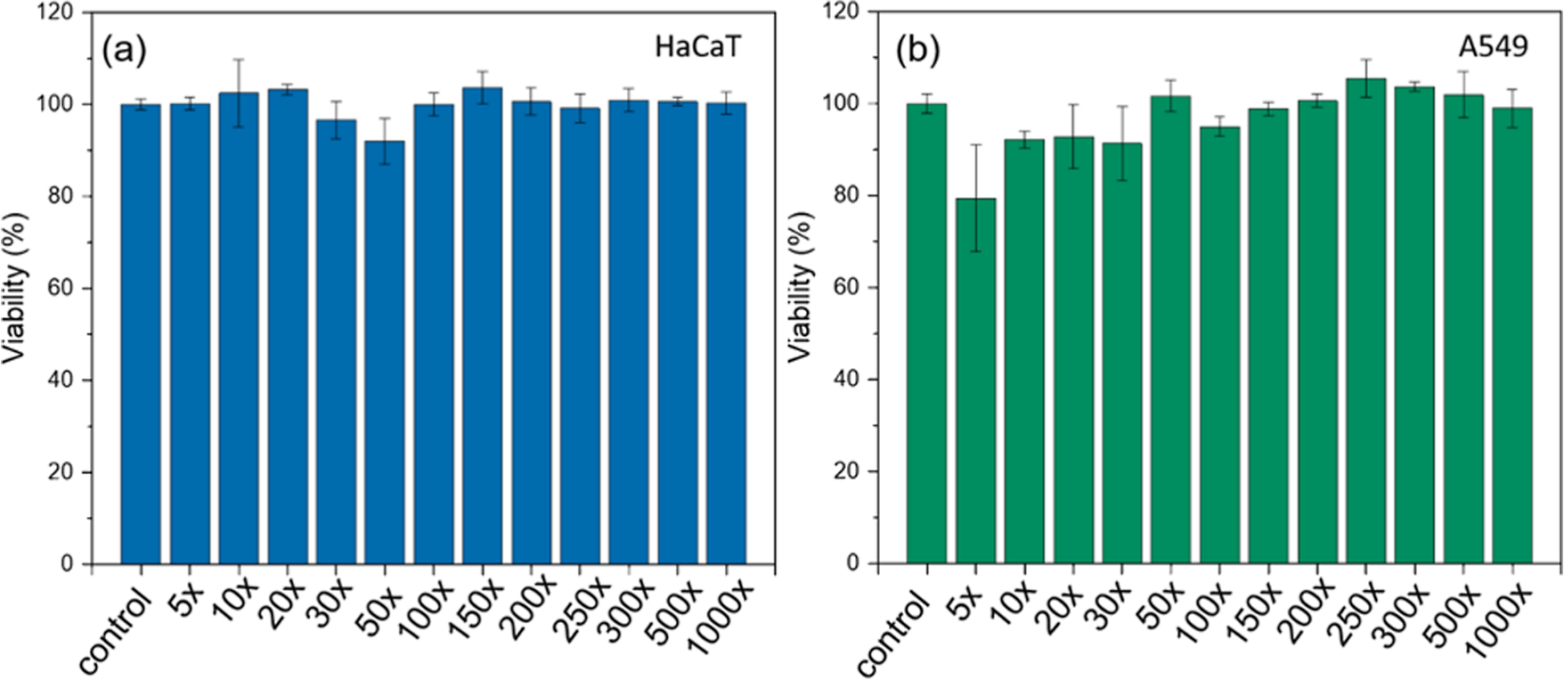
Viability of (a) HaCaT and (b) A549 cells without and with the SnO_2_NP colloid presence at different concentrations. All sample viabilities are not significantly different with *p* ≤ 0.05 in comparison to control samples.

**Figure 10:**
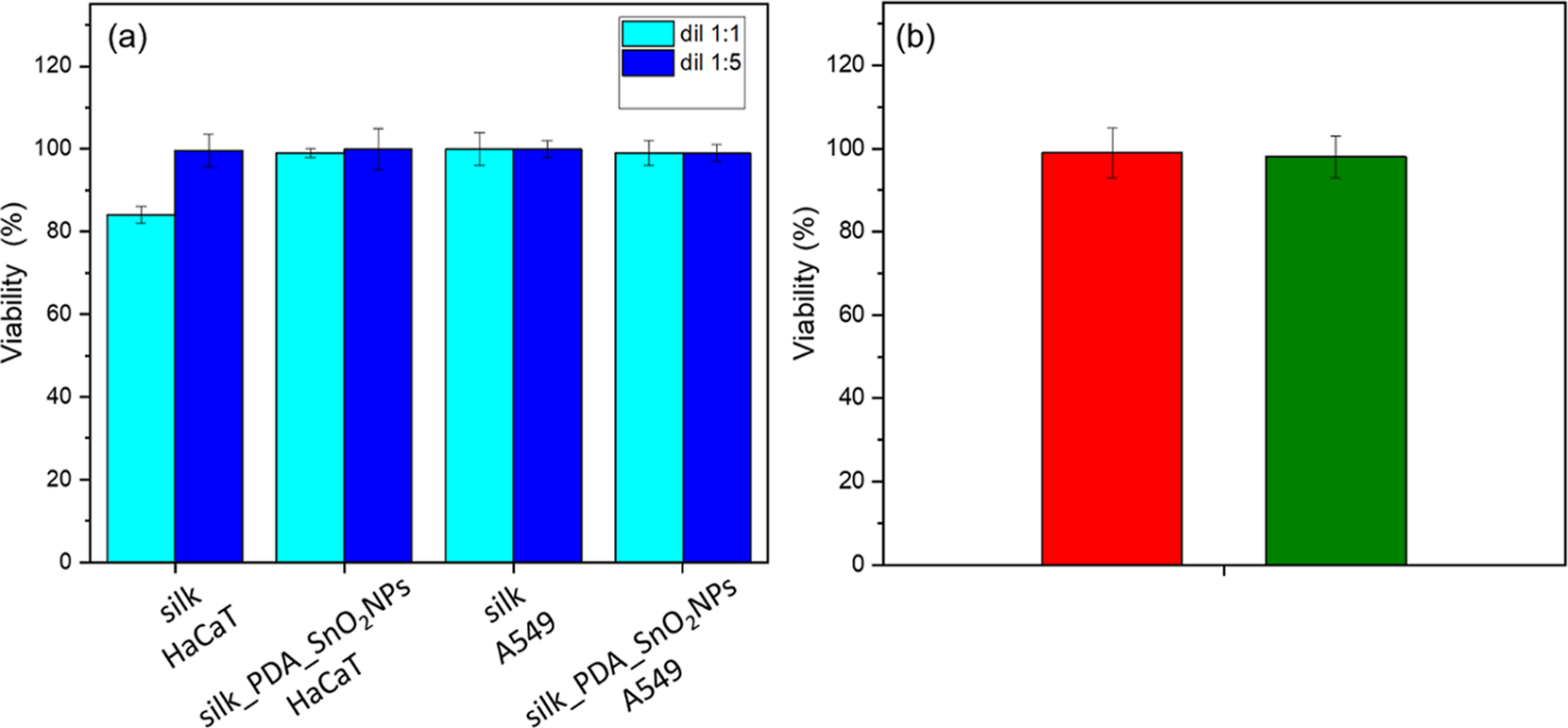
(a) Viability of HaCaT and A549 cells (WST-1 results) and (b) in vitro skin corrosion test result (WST-1, 3D skin model) with silk fabric before and after functionalization with PDA_SnO_2_NPs. (b) Viability differences are not significantly different with *p* ≤ 0.05.

Although SnO_2_NPs inhibit virus infectivity, it would be beneficial if they were biocompatible to human cells for efficient protective mask design. The cytotoxicity of SnO_2_NPs was analyzed for two different human cell lines: keratinocyte line (HaCaT), commonly used for the study of skin biology and the impact of various factors on skin, and lung carcinoma line cells (A549) to study the interaction with the respiratory tract cells. The viability of both cell lines was studied for the colloid of SnO_2_NPs (Figure 9) and silk fabric before and after the functionalization (Figure 10a).

The cytotoxicity of the SnO_2_NP colloid was evaluated after dilution from 5 to 1000 times to study the particles’ influence on cells (Figure 9). SnO_2_NPs are nontoxic in the whole range of dilution for both HaCaT and A549 cells. The viability of the cells is close to 100%, and in some cases (some dilution), it is above 100%, which means that the cells proliferate extensively regardless of the presence of the particles. This broadens SnO_2_NPs’ range of potential applications in the biological field; they can be applied to various antibacterial, antiviral, and other purposes due to their low toxicity to human cells.

The biocompatibility of SnO_2_ nanomaterials, such as nanowire coatings, was confirmed; it promotes cell proliferation and exhibits antibacterial activity.^47^ Titanium-bonding porcelain containing SnO_2_ was found to be nontoxic.^48^ The cell viability of pure silk fabric and silk fabric functionalized with PDA and SnO_2_NPs indicates their nontoxic effect (Figure 10a). Moreover, the skin corrosion test (Figure 10b) revealed that silk fabric before and after the functionalization process is not a skin-corrosive material and can be used on human skin for various purposes.

The COVID-19 pandemic has led to an increase in protective textile waste all around the world, including hazardous and plastic waste. Therefore, the aim of this study was not only to obtain a new antiviral agent but also to design a biodegradable, nontoxic, and noncorrosive protective antiviral mask to prevent the spreading of human coronaviruses and their hazardous influence as well as environmental pollution.

### 3.5 Thermophysiological Comfort of Silk Fabric Functionalized with SnO_2_NPs

**Figure 11:**
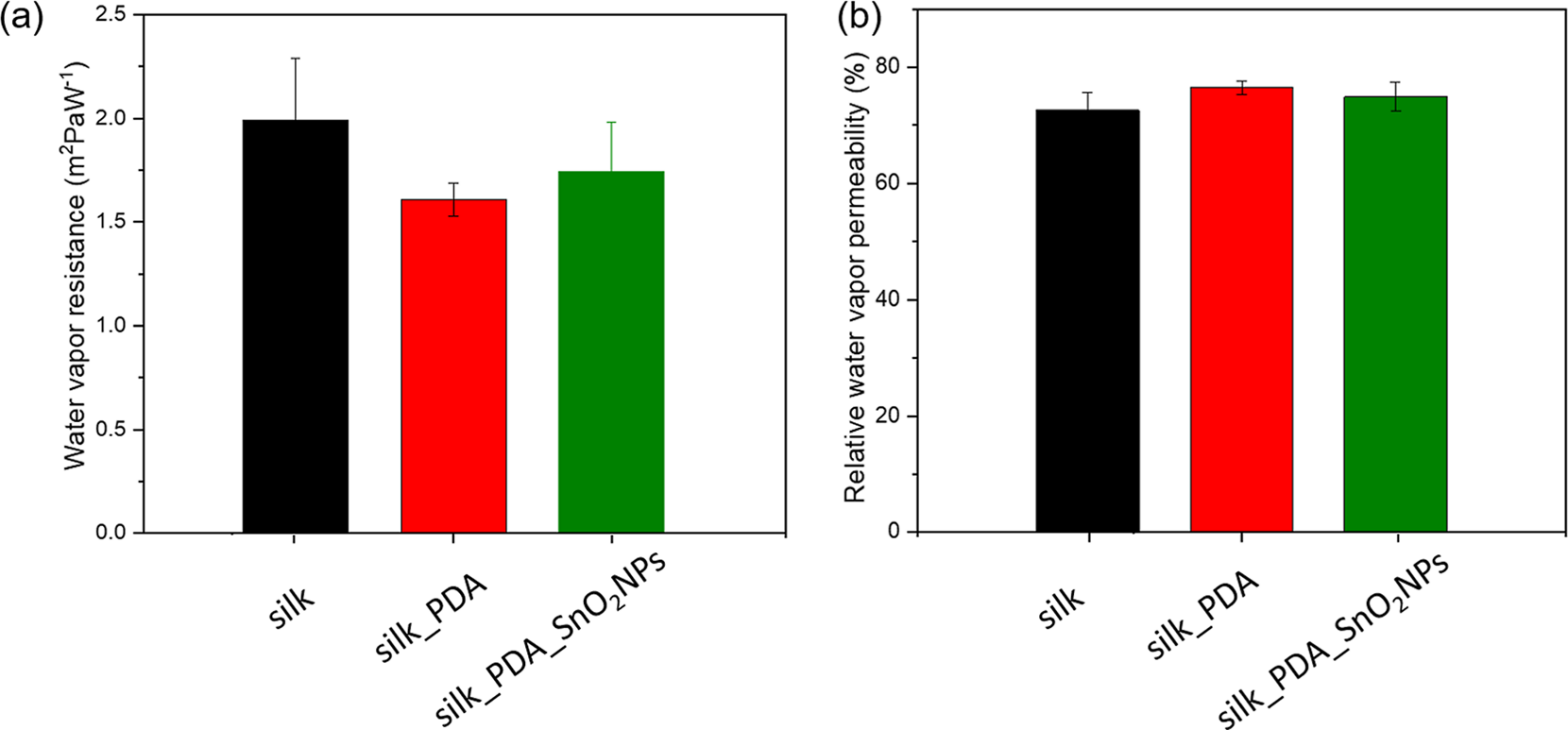
(a) Water vapor resistance and (b) relative water vapor permeability of silk fabric and silk fabric functionalized with PDA and with PDA_SnO_2_NPs.

Figure 11 shows the results of the interactions of water vapor with the fabric. The relative water vapor permeability slightly increased from 73% for silk fabric to 76.5% for silk fabric functionalized with PDA due to the high hydrophilicity of PDA. After the second functionalization step, covering PDA-coated silk fabric with SnO_2_NPs, it decreased to 75%. The ability to transmit water vapor does not change significantly. High relative water vapor permeability indicates high comfort properties and sufficient value range for protective textile structures.

**Figure 12:**
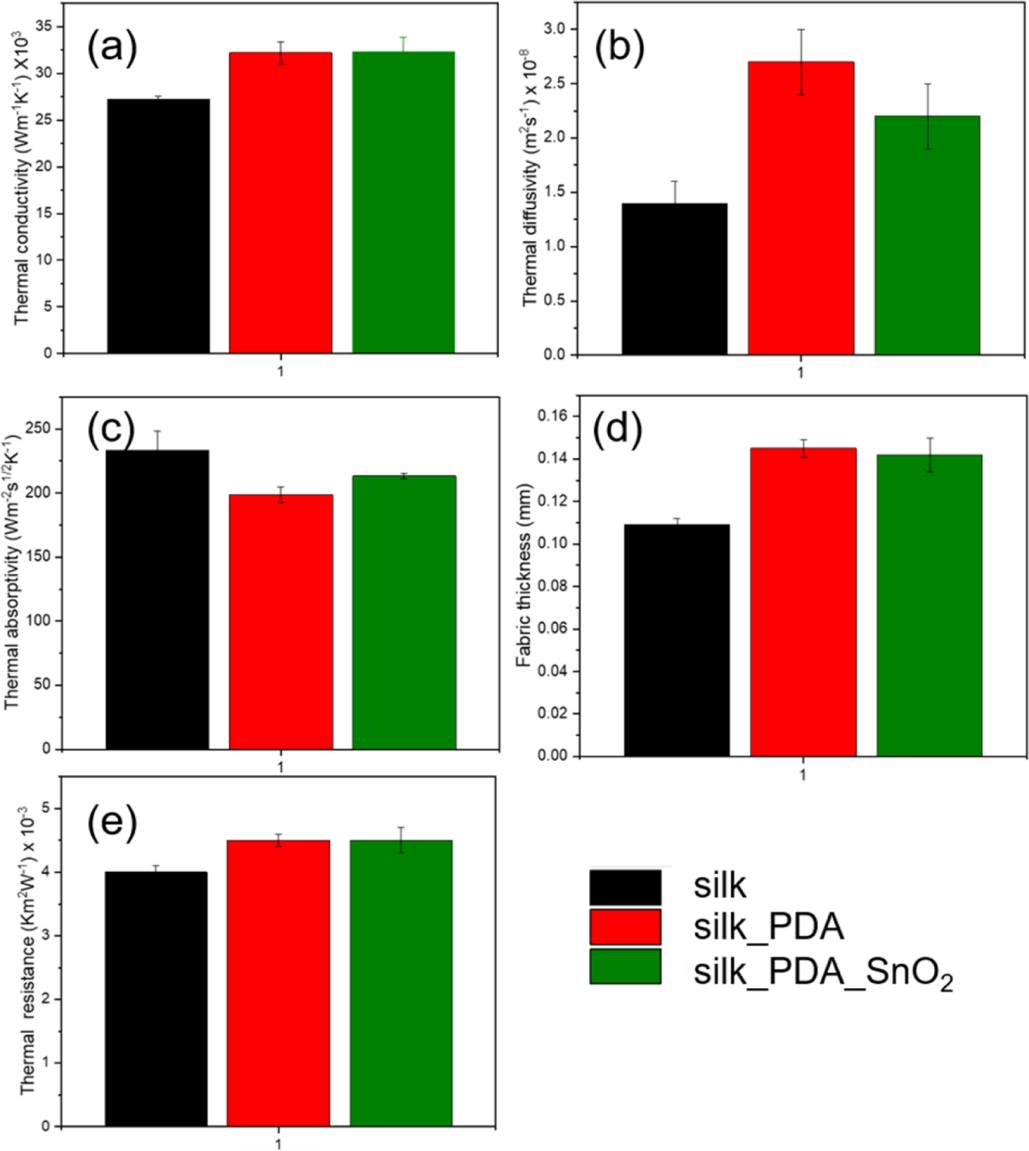
(a) Thermal conductivity, (b) thermal diffusivity, (c) thermal absorptivity, (d) thickness and (e) thermal resistance of silk fabric and silk fabric functionalized with PDA and with PDA_SnO_2_NPs.

The thermal comfort of the silk fabric after functionalization was evaluated by various measurements performed using the Alambeta device. The thermal conductivity does not change significantly (Figure 12a). It increases slightly after functionalization with PDA and SnO_2_NPs because the fabric is covered, but this effect is not considerable. The functionalization does not deteriorate the thermal comfort of the silk fabric. Thermal diffusivity describes the transport of the thermal flow through the fabric structure’s air.^49^ It slightly increases due to fabric finishing with PDA and SnO_2_NPs compared to pure silk fabric (Figure 12b). The thermal diffusivity values can clearly distinguish fabric for summer and winter clothing.^49^ The values in this study are in the range of summer clothing.^49^ It is beneficial in the case of protective mask designing because they do not cause a warming effect and feeling, avoid sweating of the skin, and allow wearing masks longer. The thermal absorptivity (Figure 12c) indicates the warm-cool feeling at the first brief contact of the fabric with the human body skin. The value of thermal absorptivity depends on finishing and fabric thickness (Figure 12d).^49^ The fabric thickness obtained from Alambeta analysis can differ from manual measurements because the device measures it automatically at the working pressure of 200 Pa using a photoelectric sensor.^50^ The thickness of the fabric with PDA is the highest, which results in the lowest value of thermal absorptivity and a greater warm feeling than pure silk fabric (Figure 12c,d). After further covering the silk fabric_PDA with SnO_2_NPs, thermal absorptivity slightly increases, reducing the warming effect of the first step of the functionalization process and providing the high thermal comfort of silk fabric with PDA and SnO_2_NPs for protective face masks. Thermal resistance (Figure 12e), which is connected with the thermal insulation of the fabric, slightly increases after functionalization but does not have a significant influence on the thermal insulation of the silk fabric. Thermal analysis of the fabric indicates that functionalization of the silk fabric with SnO_2_NPs does not deteriorate the high thermal comfort of the silk fabric and is a suitable textile for preparing protective face masks.

### 3.6 Filtration Efficiency and Breathing Resistance of Silk Fabric Functionalized with SnO_2_NPs

**Figure 13:**
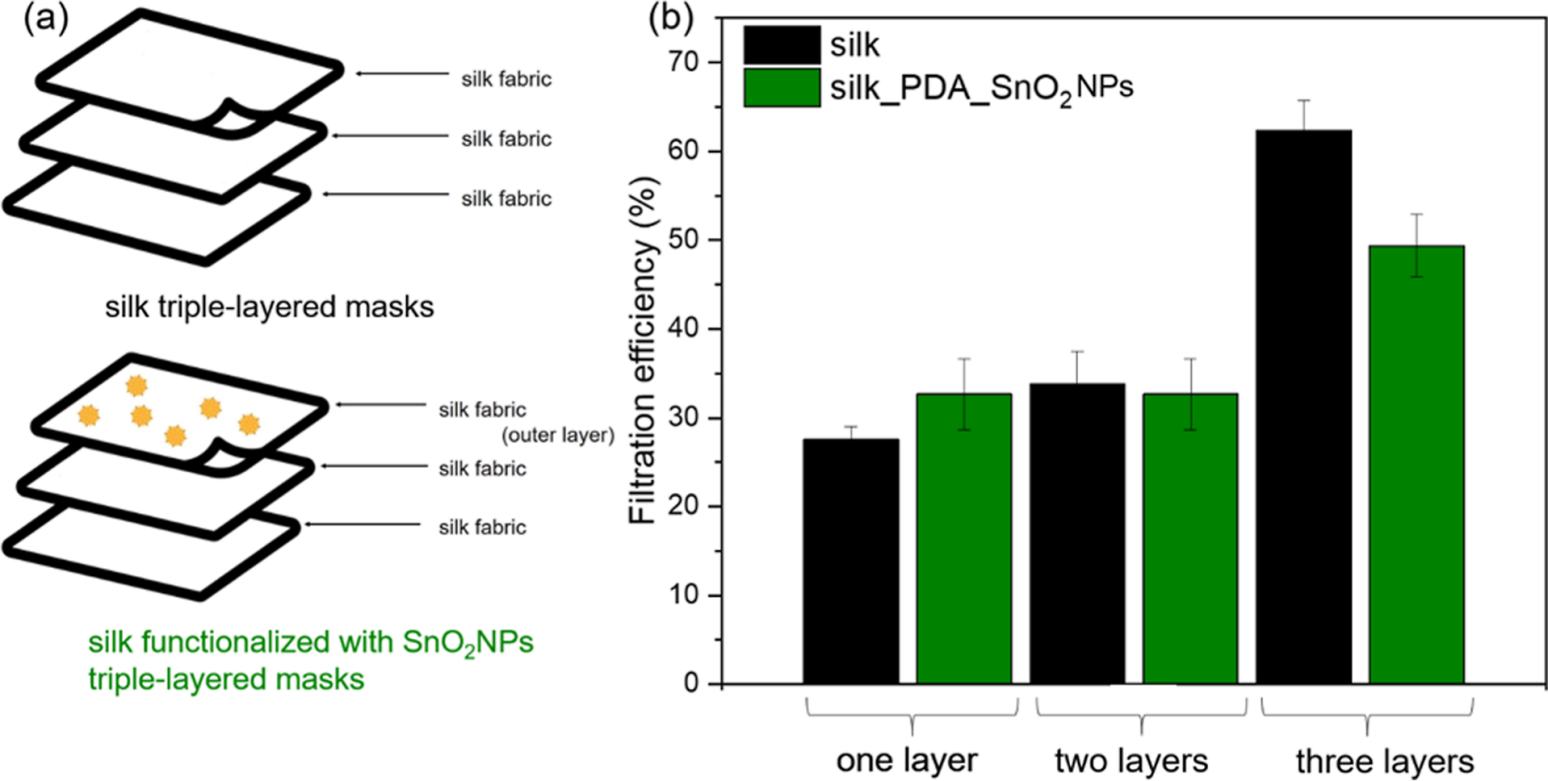
(a) Schemes of triple-layered masks and (b) filtration efficiency of silk fabric 1-, 2-, and 3-layered with the external layer made of nonfunctionalized silk and functionalized with SnO_2_NPs.

**Figure 14:**
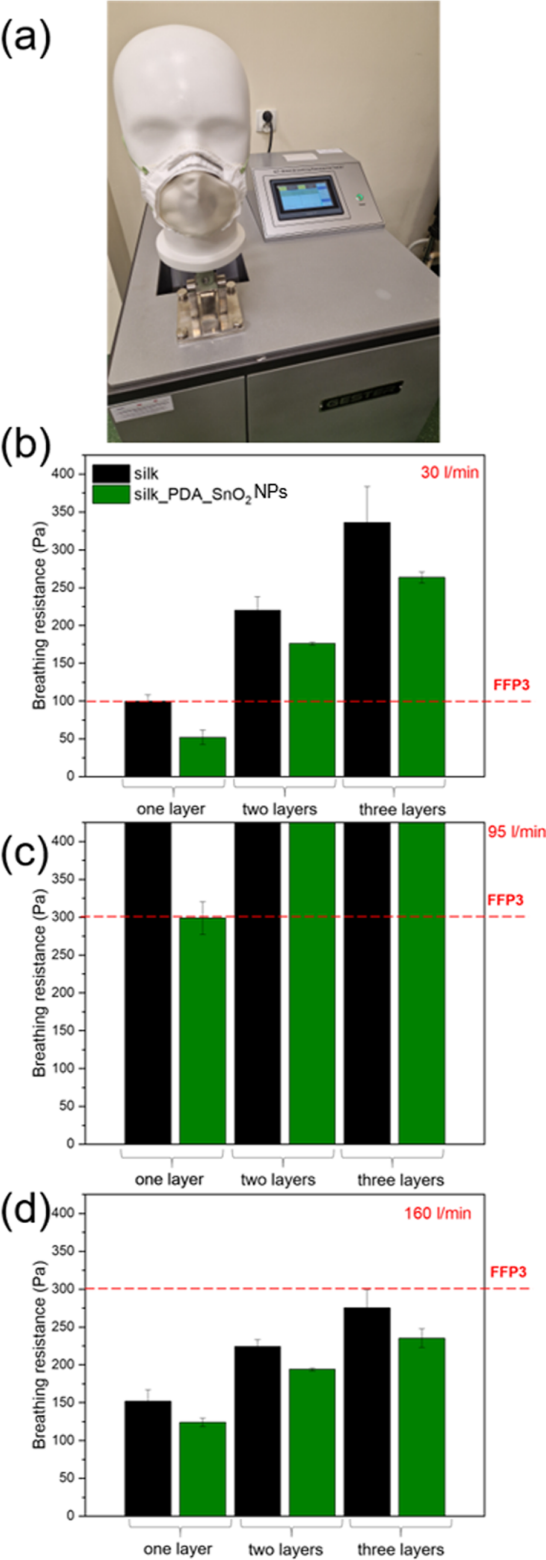
(a) GT-RA03 Mask and Respirator Breating Resistance Tester and (b,c,d) breathing resistance of silk fabric 1-, 2-, and 3-layered with the external layer made of pure silk (nonfunctionalized) and functionalized with SnO_2_NPs, measured at a flow rate of (b) 30 L/min, (c) 95 L/min, and (d) 160 L/min to imitate (b,c) inhalation and (d) exhalation.

Textile masks are commonly qualified to be protective face masks based on the measurements of filtration efficiency. The filtration efficiency was evaluated for single-, double-, and triple-layer configurations, first system composed only with pure silk and then silk fabric functionalized with SnO_2_NPs, which act as a single layer or external layer in double- and triple-layered masks (Figure 13a). The filtration efficiency increases with an increasing number of fabric layers (Figure 13b); for pure silk fabric, it was calculated to be about 28%, 34%, and 62%, respectively, for pure silk monolayer, double layer, and 3-fold layer. The same phenomenon was observed for a single silk fabric layer functionalized with SnO_2_NPs and supported with one or two pure silk fabric layers. The values of filtration efficiency for functionalized silk fabric were slightly lower due to the slight loosening of the fiber structure in the fabric after the functionalization process. The results are consistent with other reports on silk;^3,51^ the filtration efficiency for natural single silk fabric characterized by mass per unit area of about 150 g/m^2^ was about 38%,^3^ while for our fabric, 28% for more than half mass per unit area of 70 g/m^2^. The three-layer silk structure reveals an efficiency of about 60%, typical for medical masks.^3^ On the other hand, the key issue for protective mask designing is the value of breathing resistance, and it should also be taken into account. It refers to the mask’s resistance to airflow during inhalation or exhalation and describes how challenging a wearer perceives their breathing to be. In this report, breathing resistance was measured for a flow rate of 30 and 95 L/min for inhalation and 160 L/min for exhalation activity (Figure 14). The breathing resistance was higher for two- and three-layered masks. Single silk fabric functionalized with SnO_2_NPs meets the requirements for an FFP3 mask according to the European standard of EN 149:2001 + A1:2009. Although single silk fabric does not reveal sufficient filtration efficiency for high antiviral efficiency, it has such antiviral properties due to bioactive functionalization with SnO_2_NPs and resistance to airflow, allowing breathing comfort.

## 4 Conclusions

This study presents SnO_2_NPs as a novel antiviral agent against human coronavirus HCoV 229E. SnO_2_NPs were synthesized by the hydrolysis of sodium stannate and are characterized by a mean diameter of 3 nm, a cubic crystal structure, and a tendency to agglomerate. They have the capability to block virus entry, attachment, and penetration into the cell (MRC-5). SnO_2_NPs were applied to functionalize silk fabric (about 1% wt.) by using polydopamine as a linker and dip-coating process. The silk fabric functionalized with SnO_2_NPs was used to design a face-protective mask. ICP–MS analysis revealed that Sn deposition was 155 ± 30 mg/kg of the fabric.

Infrared spectroscopy showed that the functionalization process does not affect the β-sheet dominant structure of the main silk protein, silk fibroin. FTIR analysis also revealed the main bands typical for tin oxide.

Silk fabric functionalized with SnO_2_NPs reveals very good antiviral properties, above 3.13 according to the ISO18184:2019 standard. The high antiviral efficiency of SnO_2_NPs results from the negative zeta potential of the particles −28.8 (at pH 8.5), which facilitates the interaction with the positively charged virus and its deactivation. SnO_2_NPs also enhanced the hydrophobic properties of silk fabric, which is crucial for antiviral properties.

Moreover, silk fabric with SnO_2_NPs has very good antibacterial properties against Gram-positive *S. aureus* and Gram-negative *K. pneumoniae*, characterized by high bacteriostatic and biocidal effects. The reduction of both kinds of bacterial growth is above 95%. The cytotoxicity tests (WST-1) did not show the toxic effect of SnO_2_NPs in the colloid and after deposition on silk fabric. 431 Skin Corrosion test showed that functionalized silk fabric is not corrosive to human skin. The thermophysiological study confirmed the high wearing comfort of SnO_2_NP functionalized silk fabric and the possibility of using it for designing face-protective masks.

Triple-layered silk masks reveal sufficient filtration efficiency for protective textiles. In this report, it was shown that breathing resistance should also be considered when designing a face-protective mask. Only one layer of the functionalized silk fabric meets the requirements for an FFP3 mask according to the European standard of EN 149:2001 + A1:2009. One layer of the fabric covered with an efficient antiviral agent is sufficient for protection and facilitates correct breathing. It indicates a need for effective antiviral functionalization of protective textiles, not only increasing the number of layers in the mask.

The antiviral and antibacterial as well as nontoxic and not skin-corrosive properties of SnO_2_NPs make them a promising material for developing antimicrobial agents and surfaces.

## 5 Limitations

Filtration efficiency was measured using an automated filter tester (GT-RA09B-1, Gester Instruments Co., Ltd.), which is designed in a way that does not consider the impact of the particle falling out the mask itself. The individual facial contour and improper mask fit can cause small gaps between the mask material and skin,^52^ which this measurement does not consider. It may affect the real-use results of filtration efficiency. This is an efficient method for laboratory characterization and comparison of various material filtration efficiency. It should be taken into consideration before introducing the mask for use in everyday life. The leakage through the gaps around masks can significantly reduce filtration efficiency and increase the risk of infection.
